# Aneurism of the External Iliac Artery

**Published:** 1842-10-29

**Authors:** 


*Aneurism of the External Iliac Artery—Ligature of the vessel close to the Bifurcation of the common Iliac Artery.* By Mr. Bellingham.—Matthew Daly, aged thirty-two, a slender and healthy man (in other respects) was admitted into St. Vincent's Hospital, August 18, 1842, under my care, labouring under aneurism of the external iliac artery on the right side. He has never suffered from any illness of consequence; had been in the habit of drinking, but has given it up for the last two years. He is a brush-maker by trade, and his occupation consists in constantly turning a lathe with the right foot. He continued to work until three or four days previous to his admision, under the impression "that the disease would wear away."

The aneurism is of about three months duration. His attention was attracted to it by the swelling, which at that time (he says) pulsated strongly. It was, however, quite unattended by pain, and it has increased slowly in size. Recently, he has begun to suffer pain, of a dull, aching kind, in the part, which obliged him to give up work, and to seek admission into the hospital. He has never received any injury in the part, or ever strained himself that he recollects.

The aneurismal tumor is about the size of a moderately-sized orange, but more oval in shape, seated above Poupart's ligament, and extending about half an inch below it. A very strong impulse is communicated to the hand placed on it, but no fremissement. Immediately below Poupart's ligament, however, there is a slight fremissement, and on applying the stethoscope a loud single bruit is heard. There is no swelling or œdema of the foot; the circulation is perfectly regular and tranquil, and the heart's action natural.

In consultation with Sir Philip Crampton and Mr. Cusack, it was determined to place a ligature upon the common iliac artery on the right side; as it was supposed there would not be found sufficient room between the aneurism and the division of the common iliac, the external iliac being probably diseased above the aneurismal tumor.

A purgative draught was given on the night before the operation, and a cathartic enema administered the following morning, by which the bowels were well cleared out; an hour before the operation the patient took forty drops of laudanum.

The patient being placed on his back, slightly inclined to the sound side, having the thorax elevated, and the thigh bent up the pelvis, I commenced the operation (assisted by Sir Philip Crampton, Mr. Cusack, and Professor Porter) by a semi-circular incision, beginning on a line with the last rib, and terminating nearly opposite the anterior and superior spinous process of the ileum, and about an inch and a half internal to this process. Its length was about five inches, and the concavity towards the umbilicus; by this the integuments and superficial fascia were divided; the fibres of the external oblique muscle were then incised in the same direction, and next those of the internal oblique; by which the transversalis muscle was exposed. A cautious incision was made through the fibres of this muscle at the lower part of the wound, and a director endeavoured to be introduced under them. This muscle, however, was found to be considerably hypertrophied, being double or treble its natural thickness. On arriving at the transversalis fascia, it also was found to be increased considerably in thickness, and presented almost a tendinous character. A portion of it was raised with a forceps, and cautiously incised; a director was then introduced, and an incision made sufficient to admit the finger, upon which this fascia was divided by a probe-pointed bistoury, both upwards and downwards. No artery was divided in this part of the operation requiring a ligature; the bleeding appeared to be altogether venous.

The peritoneum was now very cautiously raised from the subjacent iliac muscle by insinuating the fingers behind it, and this proceeding appeared to give much more pain than was expected. As the peritoneum was detached, Mr. Porter, with his hand in the wound, drew this membrane and the intestines towards the opposite side; the separation of the peritoneum was continued until my finger reach the upper extremity of the external iliac artery; and the expected difficulty from the protrusion of the intestine was much less than had been anticipated. After a short time I succeeded in getting a view of the vessel, and, as it appeared to be perfectly healthy, its sheath was opened to a small extent by means of the blunt extremity of a director; and Mr. Trant's aneurism needle was then passed under the artery, from without inwards, without much difficulty; and here the advantage of his instrument was fully proved, for as soon as the eye of the needle appeared at the opposite side of the vessel, the ligature was drawn up by it, which, owing to the depth of the wound, and the distance of the artery from the surface, must have been attended with delay and difficulty, if the common aneurism needle had been employed. A single silk ligature was used, and as soon as it was tightened the pulsation in the aneurism ceased. The edges of the incision were then brought together by the interrupted suture and adhesive plaster; the patient was placed in bed, and took sixty drops of laudanum. The operation was completed in less than thirty-five minutes.

Eight o'clock, P. M., reaction has completely set in; both lower extremities feel warmer than natural; he has a little thirst. Pulse eighty, and has had some sleep since the operation.

August 27th.—Slept well last night; no pain any where except in the wound; no tenderness on pressure over the abdomen; no sickness of stomach: some thirst; wishes for something to eat; pulse seventy-two; temperature of the ham on right side eighty, on left ninety.

28th.—Did not rest quite so well last night; was annoyed by distension of the abdomen from flatulence, which occasionally gave him pain; pulse seventy-six; no thirst, nor pain on pressure over any part of the abdomen; the aneurismal tumor appears to be somewhat diminished in size.

26th.—Pulse seventy-two; slept comfortably last night; feels hungry, and wishes for some solid food; temperature of both limbs similar. The wound was dressed to-day; the lower portion has united by the first intention; water dressings and adhesive plaster applied; sutures not disturbed. As the bowels have not been moved since the operation, a little castor oil was directed to be administered immediately, and repeated at intervals until it operated.

30th.—Bowels moved freely; feels more comfortable since; no tension or uneasiness in the abdomen; the aneurismal tumor appears to be more solid.

31st.—Wound suppurating freely; no thirst; appetite good; temperature of both limbs equal; pulse eighty. Ordered some weak chicken broth.

September 2nd.—He complained last night of pain over the middle sternal region, increased on inspiration. A mustard cataplasm was applied, and this morning the pain is much relieved; his pulse was slightly increased in frequency, about eighty-eight; in every other respect, his condition is satisfactory.

5th.—The wound was dressed with charpie to-day; the discharge is abundant, and of a good quality; healthy granulations are springing up from the bottom; the upper and lower portion of the incision have united by the first intention; pulse regular; no thirst; appetite good; has eaten chicken for his dinner the last two days.

8th.—The wound has contracted considerably, and the discharge is diminishing; he sleeps well, and says he has not felt so well since the operation; no pulsation can be felt in the femoral or anterior tibial artery; he eats mutton chop for dinner with appetite.

13th—19th day.—The aneurismal tumor within the last day or two has become painful when pressed about the centre, and its contents at this point are evidently more fluid than before; he also suffers from a feeling of distension in the part; otherwise he is in very good health, eats his breakfast and dinner with appetite, and has been allowed porter for some days. The ligature is not yet loose, and it gives him pain when it is gently pulled.

15th—21st day.—To-day the dressings were found to be deeply coloured with blood, and, on examination, a small orifice was detected at the inferior angle of the wound, through which a mixture of pus and blood could be squeezed. The aneurismal tumor is smaller and less tense, a portion of its contents having been evacuated in this way.

16th—22nd day.—The discharge of pus and blood continues, but in diminished quantity, and the aneurismal tumor is much smaller. The ligature was gently pulled to-day, when it yielded a little. This proceeding, however, caused, apparently, very great pain, which was referred to the hip.

17th.—A feeble pulsation detected to-day in the femoral artery high up in the hip; none in the anterior tibial.

18th—24th day.—The ligature came away this morning without any pain, and was not followed by the discharge of a drop of blood. The wound is very much diminished in size, having filled up by granulation nearly to level with the skin, except at the point where the ligature presented. His health is very good; he eats heartily, and sleeps well; the aneurismal swelling is diminished in size; but the integuments at one point covering it, are discoloured and thinned, and its contents at this part are very fluid.—*Lon. Med. Gaz.* Sep. 30, 1842.

